# An in silico deep learning approach to multi-epitope vaccine design: a SARS-CoV-2 case study

**DOI:** 10.1038/s41598-021-81749-9

**Published:** 2021-02-05

**Authors:** Zikun Yang, Paul Bogdan, Shahin Nazarian

**Affiliations:** Ming Hsieh Department of Electrical and Computer Engineering, University of Southern California, Los Angeles, CA 90089 USA

**Keywords:** Peptide vaccines, Computational platforms and environments, Machine learning, Protein design, Protein function predictions

## Abstract

The rampant spread of COVID-19, an infectious disease caused by SARS-CoV-2, all over the world has led to over millions of deaths, and devastated the social, financial and political entities around the world. Without an existing effective medical therapy, vaccines are urgently needed to avoid the spread of this disease. In this study, we propose an in silico deep learning approach for prediction and design of a multi-epitope vaccine (DeepVacPred). By combining the in silico immunoinformatics and deep neural network strategies, the DeepVacPred computational framework directly predicts 26 potential vaccine subunits from the available SARS-CoV-2 spike protein sequence. We further use in silico methods to investigate the linear B-cell epitopes, Cytotoxic T Lymphocytes (CTL) epitopes, Helper T Lymphocytes (HTL) epitopes in the 26 subunit candidates and identify the best 11 of them to construct a multi-epitope vaccine for SARS-CoV-2 virus. The human population coverage, antigenicity, allergenicity, toxicity, physicochemical properties and secondary structure of the designed vaccine are evaluated via state-of-the-art bioinformatic approaches, showing good quality of the designed vaccine. The 3D structure of the designed vaccine is predicted, refined and validated by in silico tools. Finally, we optimize and insert the codon sequence into a plasmid to ensure the cloning and expression efficiency. In conclusion, this proposed artificial intelligence (AI) based vaccine discovery framework accelerates the vaccine design process and constructs a 694aa multi-epitope vaccine containing 16 B-cell epitopes, 82 CTL epitopes and 89 HTL epitopes, which is promising to fight the SARS-CoV-2 viral infection and can be further evaluated in clinical studies. Moreover, we trace the RNA mutations of the SARS-CoV-2 and ensure that the designed vaccine can tackle the recent RNA mutations of the virus.

## Introduction

Coronavirus disease 2019 (COVID-19) is an infectious disease caused by severe acute respiratory syndrome coronavirus 2 (SARS-CoV-2)^1,2^. First detected in December 2019 in Wuhan, the virus has spread globally, with basic reproduction number (R0) reaching 5.7^3^, millions of deaths, and unprecedented financial, social and political impacts all over the world^4^. Efficacious vaccines are therefore desperately needed^5^. The main clinical features of the COVID-19 are fever, cough and myalgia or fatigue^6^; the virus has caused clusters of severe respiratory illness similar to severe acute respiratory syndrome coronavirus and is associated with ICU (Intensive Care Unit) admission and high mortality rates^7^.

Currently, without a single specific antiviral therapy for SARS-CoV-2, the control methods of the COVID-19 are early diagnosis, reporting, isolation, supportive treatments, and timely publishing epidemic information with only limited impact on the coronavirus^8,9^. Researchers have proposed several approaches to develop vaccines for the SARS-CoV-2^10^. Traditional process of vaccine design is based on growing pathogens, which represents a very time-consuming process of isolating, inactivating and injecting the virus that causes the disease^11,12^. Such process usually takes more than a year to result in efficacious vaccines and hence contributes very little to avoid the current spread of the disease^13,14^. Recently, researchers have worked on constructing multi-epitope vaccines by in silico methods based on immunoinformatics without the need to grow pathogens to accelerate the vaccine design process^15–17^. Multi-epitope vaccines are constructed by multiple virus protein fragments rich in overlapping epitopes. They contain the vital part of the virus to elicit either a cellular or a humoral immune response and they reduce unwanted components that can trigger adverse effects^18^. Multi-epitope vaccines can be powerful for fighting viral infections, providing excellent vaccine candidates for clinical trials. The genome sequencing of the SARS-CoV-2 is completed^8^ and researchers have studied the details in the SARS-CoV-2 proteins^19^. Coronavirus is studded on its exterior with spike proteins, which are key components to infect and attack human cells^20^. The spike protein of the SARS-CoV-2 can latch onto cells and force the virus through the cell membrane, which enables the virus entry. Previous studies reveal that the spike protein of the SARS-CoV-2 plays a decisive role during the infection. Proteolytic activation of spike protein by host cell proteases is also a critical determinant^21^. It is promising to combat the COVID-19 by inducing the B-cells and T-cells that can perform immune responses against the SARS-CoV-2 spike protein. Hence, in this study, we choose the spike protein sequence of the SARS-CoV-2 as the main subject to design our multi-epitope vaccine.

**Figure 1:**
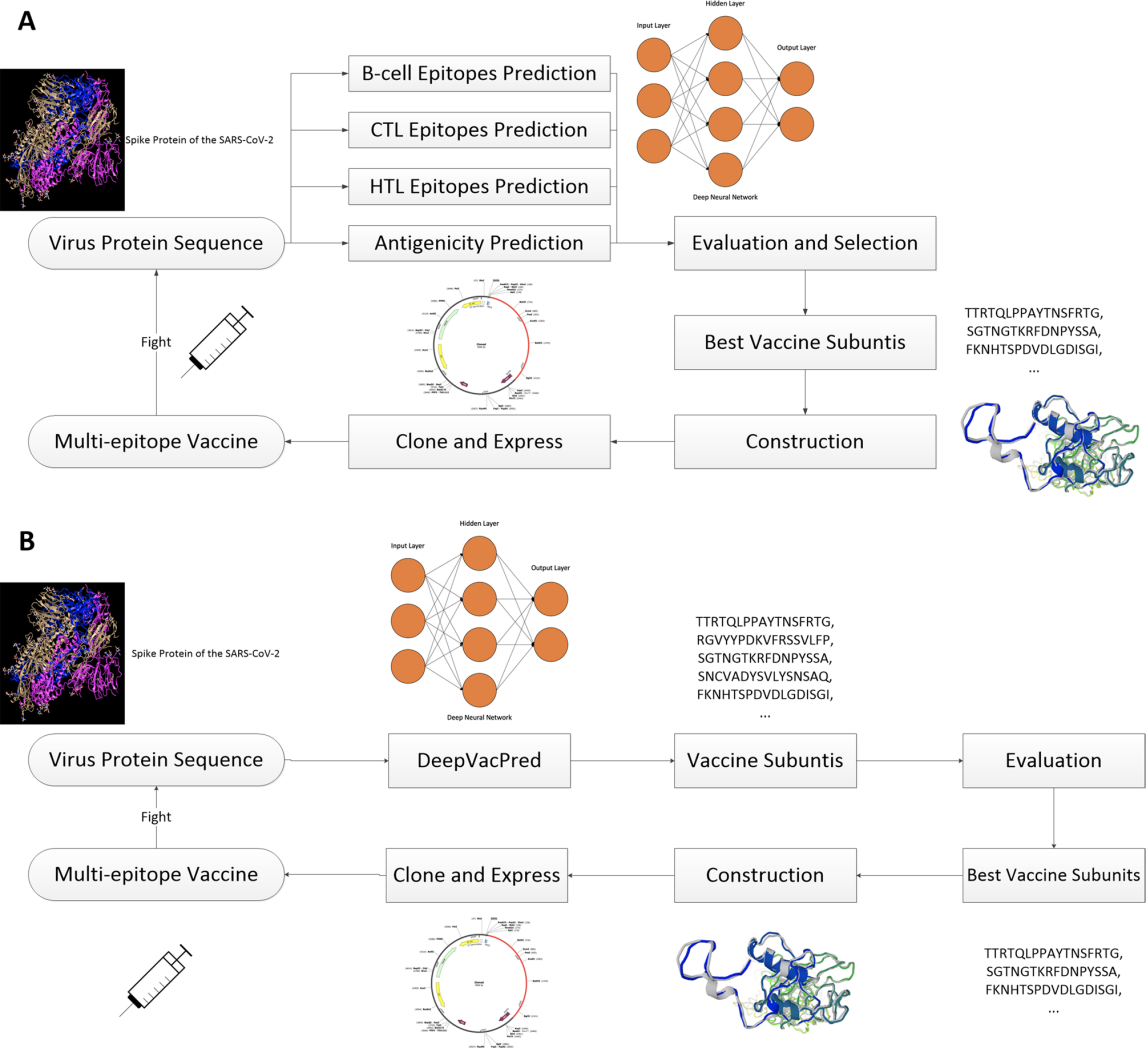
Schematic Diagram of In Silico Vaccine Design Process. **(A)** Traditional in silico vaccine design process. We have to use numerous vaccine design tools. The evaluation and subunits selection is very time consuming. No current tool is able to include all the predictions to comprehensively analyze and select out the best vaccine subunits directly. **(B)** In silico vaccine design by DeepVacPred framework. By replacing the many predictions, evaluations and selections with a DNN architecture inside the DeepVacPred framework, we are able to directly predict a very small number of potential vaccine subunits within a second and start the following evaluation and vaccine construction on a much smaller amount of data.

Although the in silico vaccine design approaches are looked at as fairly efficient, they may not be sufficiently fast to keep pace with the emergence of various pandemics. Figure 1A shows the schematic diagram of a traditional in silico vaccine design process. Researchers usually use numerous in silico tools to predict the B-cell, CTL and HTL epitopes on the whole virus proteins^22,23^. The antigenicity and other physicochemical properties of the overlapping fragments are also necessary to be evaluated^24^. To select the best virus protein regions for constructing an efficacious vaccine, we need to carefully and comprehensively evaluate all the predicted results, which creates a large overhead and can be very time consuming. Currently, each in silico vaccine design tool can only achieve one single prediction goal. For example, BepiPred^25^ is a very popular B-cell epitope prediction tool and many researchers use this tool to predict the B-cell epitopes. However, BepiPred can only be used to address the one step of B-cell epitope prediction, and when it comes to T-cell epitope prediction, a different tool such as NetMHCpan^26^ is needed. No current tool is able to conduct multiple predictions and comprehensively analyze the results for us at once to directly identify the best vaccine subunits for further construction and evaluation.

To overcome the above challenges of the in silico vaccine design, we propose DeepVacPred, a novel AI-based in silico multi-epitope vaccine design framework. We successfully replace the multiple necessary predictions and the comprehensive evaluations with a deep neural network (DNN) architecture. When the DNN takes one peptide sequence as input, it can then judge whether this input sequence can be a potential vaccine subunit. In the DeepVacPred framework, the number of potential vaccine subunits can be firstly reduced to around 30, then further evaluation and vaccine construction is done on the predicted subunits by reliable and popular in silico methods to construct the final vaccine. Our novel approach aims to achieve a much better efficiency of the in silico vaccine design.

With DeepVacPred, this study designs a multi-epitope vaccine in a novel in silico fashion. We first use the DNN architecture to lock down 26 fragments in the SARS-CoV-2 spike protein as vaccine subunit candidates. Next, we predict the linear B-cell epitopes, CTL epitopes and HTL epitopes to select and construct our final vaccine. We further analyze the human population coverage, antigenicity, allergenicity, toxicity and other physicochemical properties to validate the quality. We also predict the secondary structure and 3D structure model. This model is eventually refined and validated. Finally, the codon optimization and in silico cloning are performed to check the vaccine genome and protein constructions and ensure its effective expression. In addition, DeepVacPred allows us to quickly check for newly emerging threats caused by the RNA mutations of the SARS-CoV-2. We prove that our vaccine can tackle the virus RNA mutations.

## DeepVacPred

### Background

An in silico vaccine design process can be seen as selecting good fragments of the virus proteins, then constructing them together into a final vaccine^24^. A fragment with multiple merits can be selected as a subunit of the final vaccine. For example, an ideal subunit should contain multiple B-cell epitopes and T-cell epitopes and it should have high antigenicity to trigger human protective reactions^22,23^. These merits can be predicted by in silico approaches and currently there are numerous in silico vaccine design tools. However, these tools are designed to address only one of the several predictions at a time. Consequently, researchers have to overcome the time-consuming tasks of analyzing each individual prediction result from different tools while adopting a comprehensive view of the vaccine design. No current tool can take all the necessary merits into consideration and directly predict the vaccine subunit candidates from the virus proteins.

There are two drawbacks to the current situation: (i) We usually need only the best 10–20 subunits to construct the final vaccine while each prediction tool may provide us with hundreds or even thousands of potential locations to choose, which creates a large overhead to comprehensively select out the subunits we need and no current tool can achieve both the prediction and the selection for us. (ii) Nearly 90% prediction results are eventually discarded because they have only part of the merits, resulting in too much of unnecessary analysis and wasting many computing resources. Consequently, traditional approaches may produce vaccines that are too late or ineffective for pandemics.

In order to improve the efficiency and reliability of the vaccine design process, we improve over state-of-the-art tools by providing a DNN approach, DeepVacPred, an efficient in silico vaccine design process to address the afore-mentioned concerns. DeepVacPred directly predicts the best vaccine subunit candidates (the number is within 30) from the virus protein sequences within a second by replacing the prediction and selection with deep neural network architecture, hence promising much higher efficiencies for the vaccine design and test process.

### Data collection and dataset design

Reliable data is essential for the performance of supervised learning^27^, thus, it plays a crucial role in the outcome of the vaccine design process. We collected 5000 latest known B-cell epitopes (B) and 2000 known T-cell epitopes containing both MHC (major histocompatibility complex)-1 and MHC-2 binders^28^ (T) from the IEDB database, combining with the same number of proteins which are not T-cell or B-cell epitopes, forming a dataset of epitopes and non-epitopes. 100 known latest viral protective antigens are selected from the IEDB database, and the same number of proteins without protective functions are randomly selected, combining with the 400 antigens from previous work^29^, forming a dataset with 600 antigens.

DeepVacPred is built based on supervised learning on a subtly designed dataset. To directly predict the vaccine subunit candidates, the protein sequences in the positive dataset must contain at least one T-cell epitope and one B-cell epitope and must be protective antigens. Cartesian Product^30^ is the set that contains all ordered pairs from two sets. Thus, the two Cartesian Products, T × B and B × T, which are formed between the collected B-cell epitopes dataset and the T-cell epitopes dataset can cover all the possible combinations of the known B-cell and T-cell epitopes. We use the 600 antigens to train a neural network that can identify protective antigens. We use this neural network on the Cartesian Product to sieve out 706,970 peptides sequences that are predicted to be protective antigens. Those 706,970 peptides contain both B-cell epitopes and T-cell epitopes and are protective antigens, referred in this paper as the positive vaccine dataset. The same number of peptides randomly bridged by negative T-cell and B-cell epitopes form our negative vaccine dataset. The dataset we design addresses the three most important predictions, the B-cell epitopes, T-cell epitopes and antigenicity in the vaccine design process.

All the datasets we collected, designed and created for the DNNs training can be found in the Data Availability section. The descriptions of each dataset are shown in Table 1.

**Table 1:** Description of the datasets used for analysis and DNN training.

Datasets	Number of peptides	Descriptions
T	2000	Known T-cell epitopes with both MHC-1 and MHC-2 binders collected from the IEDB database. Used for creating the vaccine datasets
B	5000	Known B-cell epitopes collected from the IEDB database. Used for creating the vaccine datasets
Protective antigens	300	Known viral protective antigens collected from both the IEDB database and previous work. Used for training a DNN to identify protective antigens in order to sieve out the positive vaccine dataset from the Cartesian Products
Cartesian products	2000 × 5000 × 2	The Cartesian Products of TxB and BxT. The products include all the peptides generated from the T and B datasets which contain at least one T-cell epitope and one B-cell epitope in each peptide
NT	2000	2000 peptides which are not T-cell epitopes
NB	5000	5000 peptides which are not B-cell epitopes
N protective antigens	300	300 peptides which are not viral protective antigens
Positive vaccine dataset	706,970	Sieved out from the Cartesian Products by using the DNN trained by the protective antigen datasets. Each of the peptide in this dataset contains at least one T-cell epitope and one B-cell epitope and the whole sequence is predicted to be protective antigens. Used for training the DNN to predict vaccine subunits
Negative vaccine dataset	706,970	The negative dataset to train the DNN to predict vaccine subunits. Each peptide in this dataset does not contain at least one T-cell and one B-cell epitope or it is predicted to be non-protective antigens

### Network training

A multi-layer convolutional neural network (CNN) and a four-layer linear neural network connect together, forming a deep neural network (DNN) with a two-class output. The positive and negative datasets are annotated by Z-descriptors^31^, then converted to the same length of 45 vectors with auto cross covariance (ACC) transformation^32^. Trained by the transformed dataset above, the DNN achieves the classification function to predict whether the input is a protective antigen containing both the B-cell and T-cell epitopes, realizing the ability to directly judge whether a sequence can be a potential vaccine subunit. This DNN is the core part of the rapid vaccine design process of our DeepVacPred framework and we name it as DNN-V. In addition, we train another DNN with the same structure on the T-cell epitope dataset which can judge whether an input sequence can be a T-cell epitope and we name it as DNN-T. The detailed neural network structures, training process and hyper-parameters can be found in “DNN Design and Training in DeepVacPred Framework” in the Methods section.

### Validation

#### ROC curves

**Figure 2:**
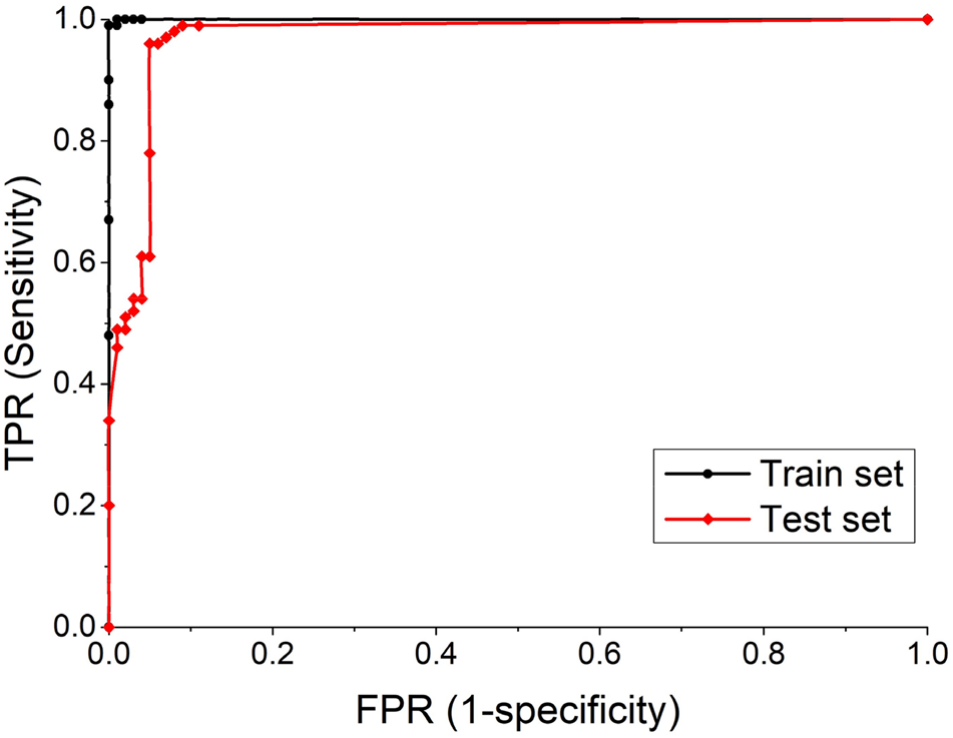
ROC Curves for the DNN-V in DeepVacPred. The area under the ROC curves represent the ability of the DNN-V to classify potential vaccine subunits and non-potential vaccine subunits. The high area under the ROC curves suggests that the DNN-V has strong classification ability and high accuracy at most threshold values.

Receiver operating characteristic (ROC) curve is a graphical plot that illustrates the diagnostic ability of a binary classifier system as its discrimination threshold is varied^33^. DNN-V is a novel approach that needs to be validated. We use the ROC curves to evaluate the DNN-V in DeepVacPred. We test the trained DNN-V with two datasets, namely the train set and the test set, each of which containing 200 protein sequences. The training set contains 200 proteins randomly selected from the dataset; we use to train the DNN-V, with 100 positive and 100 negative protein sequences. We also selected known B-cell epitopes and T-cell epitopes that are not in our collected data and use the above steps to form the testing set, also with 100 positive and 100 negative protein sequences. The ROC curves are shown in Fig. 2. The validation data appears in Table 2. The thresholds are ranged from 0 to 1. The accuracy reported in Table 2 is the greatest value among all thresholds. The sensitivity and specificity values in Table 2 are reported for the case with the highest accuracy. The AUC (Area Under the ROC Curve) value of 0.9703 for the test set which indicates the high accuracy of the classification of DNN-V to identify potential vaccine subunits.

**Table 2:** DeepVacPred Validation. For the Training Set, we reach the highest accuracy of 0.995 if the threshold value is set at 0.32. At this threshold value, the sensitivity and specificity are 0.99 each. For the Testing Set, we reach the highest accuracy of 0.95 if the threshold value is set at 0.5. At this threshold value, the sensitivity and specificity are 0.95 each. The experimental data shows high accuracy and strong classification ability of the proposed DeepVacPred framework.

Validation	AUC	Threshold	Accuracy	Sensitivity	Specificity
Train set	0.9999	0.32	0.995	0.99	0.99
Test set	0.9703	0.5	0.95	0.95	0.95

#### Vaccine design test

The false positive rate (FPR) will fall down to 0 if we set the threshold to a very low value, e.g., 0.0003, since we only care about discarding all the non-candidates. We use the DNN-V in our DeepVacPred framework on the 1273aa spike protein sequence of the SARS-CoV-2. 132 vaccine candidates are predicted. We use BepiPred^25^, NetMHCpan^26^ and Vaxijen^34^ to examine each candidate. All of the candidates contain both T-cell and B-cell epitopes and only 14 of them are predicted by Vaxijen to be non-protective antigens.

### DeepVacPred framework

Figure 1B provides the schematic diagram of the vaccine design process using DeepVacPred framework. DeepVacPred first uses DNN-V to predict a very small number of potential vaccine subunits directly from the virus protein sequences. DeepVacPred further uses DNN-T to examine all the overlapping sequences in these subunits and select the subunit candidates which have multiple T-cell epitopes. These two prediction rounds take less than 1 s and reduce the number of potential vaccine subunits to around 30. Compared to traditional approaches, the most time-consuming subunits selection part can be easily done by DeepVacPred within less than a second, saving a large amount of time and computational resources.

The following steps in the DeepVacPred framework are as follows: (i) selecting the best subunits from only about 30 candidates and (ii) constructing the final vaccine based on the evaluations by various reliable in silico tools, including Linear B-cell epitopes prediction, CTL and HTL epitopes prediction, population coverage analysis, vaccine construction, evaluation of antigenicity, allergenicity, solubility, immunogenicity, toxicity and other physicochemical properties, structure prediction, 3D modeling, in silico cloning, molecular docking and molecular dynamics simulation. Compared to the popular computational process, those evaluations are done on a much smaller amount of data, hence improving the efficiency.

## Results

### Data retrieval

The genome sequence of SARS-CoV-2 isolate Wuhan-Hu-1 is retrieved from the NCBI database with accession number MN908947^35^. The protein sequences are retrieved according to their translation. Especially, the spike protein (protein ID: QHD43416.1) has a length of 1273 amino acids (aa), and the receptor binding domain (RBD) is from 347 to 520aa^20^. The following experiments are mainly focused on the spike protein region.

### DeepVacPred vaccine subunits prediction

All the overlapping protein fragments with a length of 30aa are generated out of the 1273aa SARs-CoV-2 spike protein sequence. DeepVacPred first tests these 1244 30aa protein sequences and predicts 132 potential vaccine subunits (see Table 3). The DeepVacPred framework further predicts the T-cell epitopes at these locations and discards the subunits which have less than 8 T-cell epitopes^36^. After this prediction, our DeepVacPred provides us with 26 potential vaccine subunits for further evaluation and construction (see Table 4). These subunits are very likely to contain B-cell epitopes and multiple T-cell epitopes. They are also very likely to have high antigenicity and low allergenicity. We start the following in silico vaccine design process directly from the predicted 26 vaccine subunits, which is very efficient.

**Table 3:** DeepVacPred first round prediction results. Here we show the number of predicted vaccine subunits for each location.

Location	Proteins	Start	End	Number of vaccine subunits
Location 1	Spike	6	36	2
Location 2	Spike	53	104	3
Location 3	Spike	105	167	8
Location 4	Spike	206	322	22
Location 5	Spike	352	585	30
Location 6	Spike	601	741	19
Location 7	Spike	751	862	17
Location 8	Spike	878	981	16
Location 9	Spike	1034	1063	1
Location 10	Spike	1057	1186	12
Location 11	Spike	1188	1218	2

**Table 4:** DeepVacPred second round prediction results. Here we get 26 vaccine subunits for further evaluation and construction. Those 26 vaccine subunits are very likely to have high antigenicity and contain multiple B-cell and T-cell epitopes. With DeepVacPred, those 26 vaccine subunits are reached within less than a second, while it can take days to select those subunits from the virus protein if we use traditional methods. Next, DeepVacPred simply checks the epitopes and other merits on those 26 subunits and constructs the multi-epitope vaccine directly from those 26 candidates, which is much more efficient than traditional approaches.

Vaccine subunits	Protein	Start	End	Peptide sequence
Subunit 1	Spike	19	48	TTRTQLPPAYTNSFTRGVYYPDKVFRSSVL
Subunit 2	Spike	34	63	RGVYYPDKVFRSSVLHSTQDLFLPFFSNVT
Subunit 3	Spike	71	100	SGTNGTKRFDNPVLPFNDGVYFASTEKSNI
Subunit 4	Spike	141	170	LGVYYHKNNKSWMESEFRVYSSANNCTFEY
Subunit 5	Spike	191	220	FVFKNIDGYFKIYSKHTPINLVRDLPQGFS
Subunit 6	Spike	209	238	PINLVRDLPQGFSALEPLVDLPIGINITRF
Subunit 7	Spike	306	335	FTVEKGIYQTSNFRVQPTESIVRFPNITNL
Subunit 8	Spike	359	388	SNCVADYSVLYNSASFSTFKCYGVSPTKLN
Subunit 9	Spike	402	431	IRGDEVRQIAPGQTGKIADYNYKLPDDFTG
Subunit 10	Spike	439	468	NNLDSKVGGNYNYLYRLFRKSNLKPFERDI
Subunit 11	Spike	480	509	CNGVEGFNCYFPLQSYGFQPTNGVGYQPYR
Subunit 12	Spike	510	539	VVVLSFELLHAPATVCGPKKSTNLVKNKCV
Subunit 13	Spike	584	613	ILDITPCSFGGVSVITPGTNTSNQVAVLYQ
Subunit 14	Spike	626	655	ADQLTPTWRVYSTGSNVFQTRAGCLIGAEH
Subunit 15	Spike	655	684	HVNNSYECDIPIGAGICASYQTQTNSPRRA
Subunit 16	Spike	697	726	MSLGAENSVAYSNNSIAIPTNFTISVTTEI
Subunit 17	Spike	709	738	NNSIAIPTNFTISVTTEILPVSMTKTSVDC
Subunit 18	Spike	773	802	EQDKNTQEVFAQVKQIYKTPPIKDFGGFNF
Subunit 19	Spike	805	834	LPDPSKPSKRSFIEDLLFNKVTLADAGFIK
Subunit 20	Spike	866	895	TDEMIAQYTSALLAGTITSGWTFGAGAALQ
Subunit 21	Spike	946	975	GKLQDVVNQNAQALNTLVKQLSSNFGAISS
Subunit 22	Spike	1017	1046	EIRASANLAATKMSECVLGQSKRVDFCGKG
Subunit 23	Spike	1034	1063	LGQSKRVDFCGKGYHLMSFPQSAPHGVVFL
Subunit 24	Spike	1094	1123	VFVSNGTHWFVTQRNFYEPQIITTDNTFVS
Subunit 25	Spike	1156	1185	FKNHTSPDVDLGDISGINASVVNIQKEIDR
Subunit 26	Spike	1179	1208	IQKEIDRLNEVAKNLNESLIDLQELGKYEQ

### Linear B-cell epitopes prediction

**Figure 3:**
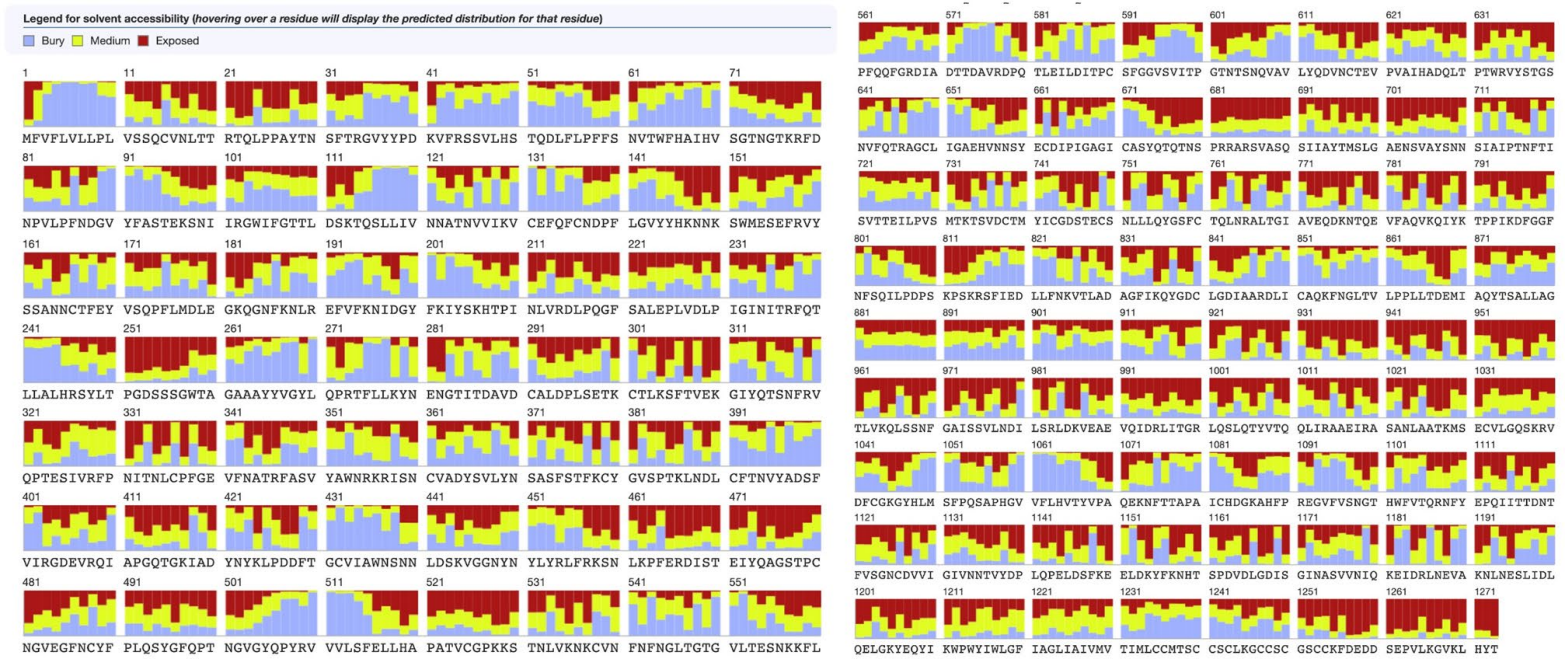
Surface accessibility of the SARS-CoV-2. The red color represents the exposed residues, the yellow color represents the medium exposed residues and the blue color represents the buried residues. In the SARS-CoV-2 spike protein, the B-cell epitopes in the 14 vaccine subunits are well-exposed according to the surface accessibility prediction, showing good potential that the B-cell receptor is able to interact with the virus to trigger the immune response.

B-cell epitopes are portions of antigens binding to immunoglobulin or antibody to trigger the B-cells to provide immune response^37^. Linear B-cell epitopes are predicted on the 26 vaccine subunits. Linear B-cell epitopes are predicted by four online servers including BepiPred^25^, SVMtrip^38^, ABCPred^39^ and BCPreds^40^. We first use BepiPred for the main prediction and we use the other three servers to check the prediction results by BepiPred. A B-cell epitope predicted by the BepiPred will be discarded if it is not predicted by any of the other three servers. B-cell epitopes must be located in the solvent-exposed region of the antigens to be possible to combine with the B-cell^37^, thus it is essential to predict the surface availability of the structural protein sequence. The surface availability is predicted by Emini tool^41,42^ on the whole SARS-CoV-2 spike protein sequence, and we discarded the epitopes that are not exposed on the surface. After the predictions, we select out 14 vaccine subunits (see Table 5). We further use the RaptorX Property server to evaluate the surface accessibility of the SARS-CoV-2 to validate that the B-cell epitopes in those subunits are well-exposed (see Fig. 3).

**Table 5:** Linear B-cell Epitopes Prediction Results. Here, we show the selected 14 vaccine subunits, the contained B-cell epitopes and their Emini scores.

Vaccine Subunits	Protein	Start	End	Peptide Sequence	B-cell Epitopes	Emini Score
Subunit 1	Spike	19	48	TTRTQLPPAYTNSFTRGVYYPDKVFRSSVL	TTRTQLPPAYTNSF	1.937
Subunit 3	Spike	71	100	SGTNGTKRFDNPVLPFNDGVYFASTEKSNI	NGTKRFD	2.678
					KSNI	1.395
Subunit 4	Spike	141	170	LGVYYHKNNKSWMESEFRVYSSANNCTFEY	YYHKNNKS	3.544
Subunit 5	Spike	191	220	FVFKNIDGYFKIYSKHTPINLVRDLPQGFS	HTPIN	1.207
Subunit 9	Spike	402	431	IRGDEVRQIAPGQTGKIADYNYKLPDDFTG	EVRQIAPGQTGKIADYNYK	1.775
Subunit 10	Spike	439	468	NNLDSKVGGNYNYLYRLFRKSNLKPFERDI	NNLDSKV	1.508
					LFRKSN	2.403
Subunit 13	Spike	584	613	ILDITPCSFGGVSVITPGTNTSNQVAVLYQ	GTNTSN	1.888
Subunit 15	Spike	655	684	HVNNSYECDIPIGAGICASYQTQTNSPRRA	HVNNSY	1.460
					YQTQTNSPRRAR	3.849
Subunit 18	Spike	773	802	EQDKNTQEVFAQVKQIYKTPPIKDFGGFNF	QDKNTQ	4.752
					KQIYKTPPI	2.243
Subunit 19	Spike	805	834	LPDPSKPSKRSFIEDLLFNKVTLADAGFIK	LPDPSKPSKR	3.136
Subunit 23	Spike	1034	1063	LGQSKRVDFCGKGYHLMSFPQSAPHGVVFL	GQSKRVDFC	1.098
					FPQSAPH	1.001
Subunit 24	Spike	1094	1123	VFVSNGTHWFVTQRNFYEPQIITTDNTFVS	FYEPQIITTD	1.627
Subunit 25	Spike	1156	1185	FKNHTSPDVDLGDISGINASVVNIQKEIDR	DKYFKNHTSPDVDLGDIS	1.833
					IQKEIDR	1.666
Subunit 26	Spike	1179	1208	IQKEIDRLNEVAKNLNESLIDLQELGKYEQ	IQKEIDR	1.666
					ELGKY	2.802

### Cytotoxic T lymphocytes (CTL) epitopes prediction

Cytotoxic T Lymphocytes (CTL) recognize the infected cells by using the MHC class I molecules to bind with certain CTL epitopes^26^. We use NetMHCpan 4.1 server^43^ to predict potential CTL epitopes. All the overlapping 9aa peptide sequences in the 14 vaccine subunits are tested with the most common 12 human-leukocyte-antigen (HLA) Class I alleles including HLA-A1, HLA-A2, HLA-A3, HLA-A24, HLA-A26, HLA-B7, HLA-B8, HLA-B27, HLA-B39, HLA-B44, HLA-B58 and HLA-B62 to evaluate their binding affinities and predict potential CTL epitopes^26,44^. The total HLA score is calculated for each vaccine subunits. The results are shown in Table 6.

**Table 6:** CTL epitopes prediction results.

Subunits	Peptide sequence	CTL epitopes	HLA class I alleles and supertypes	HLA score
Subunit 1	TTRTQLPPAYTNSFTRGVYYPDKVFRSSVL	9	A1, A2, A24, A26, B7, B8, B27, B39, B58, B62	4.652
Subunit 3	SGTNGTKRFDNPVLPFNDGVYFASTEKSNI	6	A1, A3, A24, B7, B27, B39, B62	2.492
Subunit 4	LGVYYHKNNKSWMESEFRVYSSANNCTFEY	9	A1, A3, A24, A26, B39, B40, B58, B62	6.124
Subunit 5	FVFKNIDGYFKIYSKHTPINLVRDLPQGFS	9	A1, A2, A24, A26, B7, B8, B27, B39, B58, B62	7.131
Subunit 9	IRGDEVRQIAPGQTGKIADYNYKLPDDFTG	6	A2, A3, B7, B27, B62	3.092
Subunit 10	NNLDSKVGGNYNYLYRLFRKSNLKPFERDI	9	A1, A3, A24, B8, B27, B39, B62	4.326
Subunit 13	ILDITPCSFGGVSVITPGTNTSNQVAVLYQ	5	A1, A3, A24, B8, B27, B39, B62	5.837
Subunit 15	HVNNSYECDIPIGAGICASYQTQTNSPRRA	3	A1, B7, B40, B62	0.211
Subunit 18	EQDKNTQEVFAQVKQIYKTPPIKDFGGFNF	7	A1, A2, A3, A24, A26, B8, B39, B40, B62	4.282
Subunit 19	LPDPSKPSKRSFIEDLLFNKVTLADAGFIK	8	A1, A2, A3, A24, B7, B8, B27, B39, B40, B58, B62	5.763
Subunit 23	LGQSKRVDFCGKGYHLMSFPQSAPHGVVFL	8	A1, A2, A3, A24, A26, B7, B8, B39, B58, B62	6.167
Subunit 24	VFVSNGTHWFVTQRNFYEPQIITTDNTFVS	8	A2, A3, A24, A26, B27, B39, B58, B62	5.66
Subunit 25	FKNHTSPDVDLGDISGINASVVNIQKEIDR	4	A2, A26, B39	1.341
Subunit 26	IQKEIDRLNEVAKNLNESLIDLQELGKYEQ	5	A1, A2, B7, B8, B40, B62	3.26

### Helper T lymphocytes (HTL) epitopes prediction

Helper T Lymphocytes (HTL) help the activity of other immune cells and they recognize the infection by using MHC class II molecules to bind with certain HTL epitopes^45^. We use NetMHCIIpan 4.0 server^46^ to predict potential HTL epitopes. All the overlapping 15aa peptide sequences in the 14 vaccine subunits are tested with the most common 13 HLA Class II alleles including HLA-DRB1-0101, HLA-DRB1-0301, HLA-DRB1-0401, HLA-DRB1-0701, HLA-DRB1-0801, HLA-DRB1-0901, HLA-DRB1-1001, HLA-DRB1-1101, HLA-DRB1-1201, HLA-DRB1-1301, HLA-DRB1-1401, HLA-DRB1-1501, HLA-DRB1-1601 to evaluate their binding affinities and predict the potential HTL epitopes^45,47^. The total HLA score is calculated for each vaccine subunits. The results appears in Table 7.

**Table 7:** HTL epitopes prediction results.

Subunits	Peptide sequence	HTL epitopes	HLA class II (HLA-DRB1*:01) alleles	HLA score
Subunit 1	TTRTQLPPAYTNSFTRGVYYPDKVFRSSVL	9	01, 03, 04, 07, 08, 09, 10, 11, 13, 15, 16	18.031
Subunit 3	SGTNGTKRFDNPVLPFNDGVYFASTEKSNI	10	01, 04, 07, 08, 09, 10, 12, 13, 14, 15	9.07
Subunit 4	LGVYYHKNNKSWMESEFRVYSSANNCTFEY	9	04, 08, 10, 11, 13, 15, 16	7.38
Subunit 5	FVFKNIDGYFKIYSKHTPINLVRDLPQGFS	14	01, 03, 04, 07, 08, 09, 10, 11, 12, 13, 14, 15, 16	26.785
Subunit 9	IRGDEVRQIAPGQTGKIADYNYKLPDDFTG	7	01, 07, 09, 10, 14	4.932
Subunit 10	NNLDSKVGGNYNYLYRLFRKSNLKPFERDI	8	07, 08, 11, 13, 14, 16	12.14
Subunit 13	ILDITPCSFGGVSVITPGTNTSNQVAVLYQ	2	10	0.618
Subunit 15	HVNNSYECDIPIGAGICASYQTQTNSPRRA	4	01, 03, 04, 09, 10, 16	3.986
Subunit 18	EQDKNTQEVFAQVKQIYKTPPIKDFGGFNF	9	03, 04 ,07, 08, 09, 10, 11, 12, 13, 14, 15, 16	21.858
Subunit 19	LPDPSKPSKRSFIEDLLFNKVTLADAGFIK	8	03, 04, 08, 09, 10, 11, 14	5.479
Subunit 23	LGQSKRVDFCGKGYHLMSFPQSAPHGVVFL	4	01, 04, 08, 10, 11	2.996
Subunit 24	VFVSNGTHWFVTQRNFYEPQIITTDNTFVS	8	03, 04, 07, 08, 09, 10, 11, 12, 13, 14, 15, 16	11.56
Subunit 25	FKNHTSPDVDLGDISGINASVVNIQKEIDR	8	01, 04, 07, 08, 09, 10, 11, 12, 13, 14, 15	11.925
Subunit 26	IQKEIDRLNEVAKNLNESLIDLQELGKYEQ	6	08, 11, 12, 14	3.489

### Worldwide human population coverage analysis

The vaccine we design should have wide human population coverage. We use the IEDB population coverage analysis tool^48^ to evaluate the worldwide human population coverage of the 14 vaccine subunits. The 25 HLA alleles we used to predict the T-cell epitopes can cover 98.39% of the human population. The human population coverage of each vaccine subunit is shown in Table 8. The results suggest that our 14 vaccine subunits can cover a very wide range of human population.

**Table 8:** Worldwide human population coverage analysis results.

Vaccine subunits	Protein	Start	End	Peptide sequence	Population coverage (worldwide) %
Subunit 1	Spike	19	48	TTRTQLPPAYTNSFTRGVYYPDKVFRSSVL	96.95
Subunit 3	Spike	71	100	SGTNGTKRFDNPVLPFNDGVYFASTEKSNI	83.02
Subunit 4	Spike	141	170	LGVYYHKNNKSWMESEFRVYSSANNCTFEY	81.74
Subunit 5	Spike	191	220	FVFKNIDGYFKIYSKHTPINLVRDLPQGFS	97.04
Subunit 9	Spike	402	431	IRGDEVRQIAPGQTGKIADYNYKLPDDFTG	77.19
Subunit 10	Spike	439	468	NNLDSKVGGNYNYLYRLFRKSNLKPFERDI	78.51
Subunit 13	Spike	584	613	ILDITPCSFGGVSVITPGTNTSNQVAVLYQ	61.44
Subunit 15	Spike	655	684	HVNNSYECDIPIGAGICASYQTQTNSPRRA	68.94
Subunit 18	Spike	773	802	EQDKNTQEVFAQVKQIYKTPPIKDFGGFNF	90.19
Subunit 19	Spike	805	834	LPDPSKPSKRSFIEDLLFNKVTLADAGFIK	76.12
Subunit 23	Spike	1034	1063	LGQSKRVDFCGKGYHLMSFPQSAPHGVVFL	68.38
Subunit 24	Spike	1094	1123	VFVSNGTHWFVTQRNFYEPQIITTDNTFVS	94.90
Subunit 25	Spike	1156	1185	FKNHTSPDVDLGDISGINASVVNIQKEIDR	87.47
Subunit 26	Spike	1179	1208	IQKEIDRLNEVAKNLNESLIDLQELGKYEQ	76.72

### Multi-epitope vaccine construction

**Figure 4:**
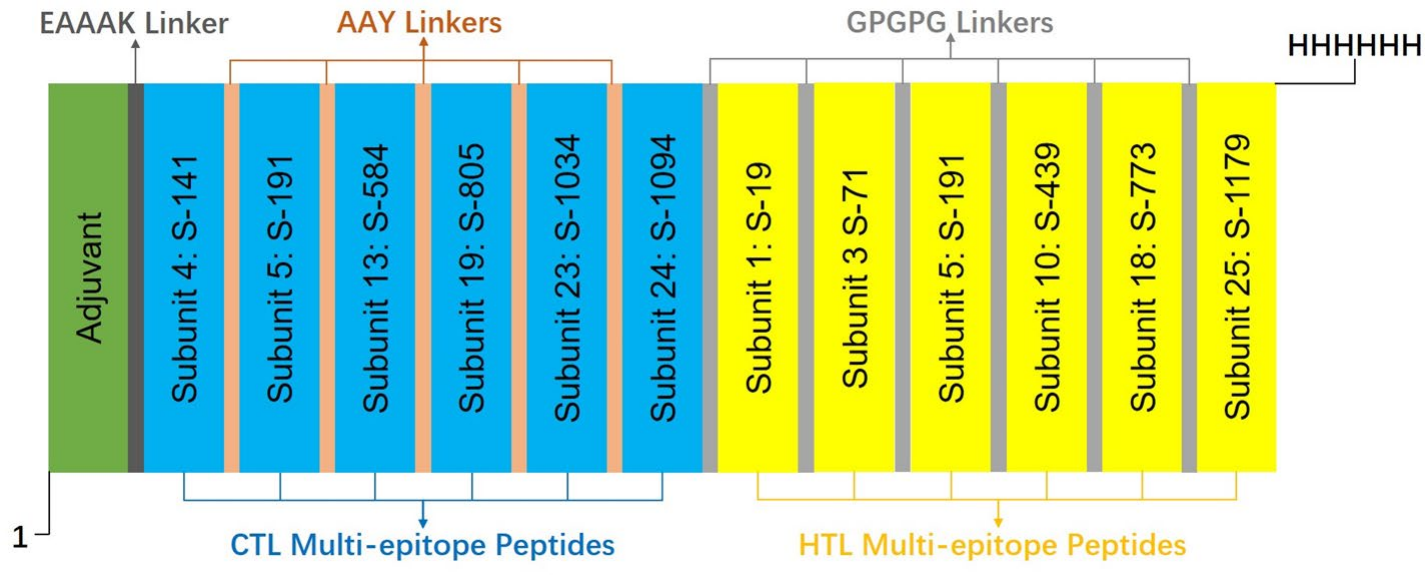
Schematic Presentation of the final Multi-epitope Vaccine. The vaccine is constructed by 11 subunits (Subunit 5 is used twice in both CTL and HTL region for its good performance), an adjuvant and a 6xHis tag, linked by EAAAK, AAY and GPGPG linkers. The final vaccine consists of 694 amino acid residues. It contains 16 B-cell epitopes, 82 CTL epitopes and 89 HTL epitopes.

We discard Subunits 9, 15 and 26 for their poor performance in the CTL and HTL epitope predictions. We use the remaining 11 vaccine subunits to construct a final multi-epitope vaccine (see Fig. 4). To avoid potential autoimmunity, we perform a BLASTp screening against the Uniprot database on those 11 vaccine subunits. A subunit with a higher-than-35% identity will be considered as homologous protein with human proteome. Among the 11 vaccine subunits we choose for the final vaccine construction, none of them show high degree of homology with the human proteome. The final vaccine contains an adjuvant, 50S ribosomal protein L2^49,50^ (accession no. AXI95322.1), to improve the immune response^51^, linked with the amino (N) terminum of the multi-subunit sequence through an EAAAK linker^52^. The multi-subunit sequence has a CTL multi-epitope peptides region followed by an HTL multi-epitope peptides region. The CTL region is constructed by 6 subunits which have better performance in the CTL epitopes prediction. AAY linkers^52^ are used in this region to fuse the subunits. The HTL region is constructed by 6 subunits which have better performance in the HTL epitopes prediction. GPGPG linkers^52^ are used in this region to fuse the subunits. The two regions are linked through a GPGPG linker. In addition, Subunit 5 is used twice in both CTL and HTL region for its good performance in both CTL and HTL epitope predictions. In the end, a 6xHis tag is added at the C-terminal to help purify and identify the protein^53^. The final vaccine consists of 694 amino acid residues. It contains 16 B-cell epitopes, 82 CTL epitopes and 89 HTL epitopes.

### Antigenicity, allergenicity and solubility evaluation

The antigenicity of the final multi-epitope vaccine sequence is evaluated by the Vaxijen 2.0 online server^34,54^ and the AntigenPro server^55^. We also evaluate the antigenicity of each vaccine subunit, including the adjuvant (see Table 9). The Vaxijen score for the whole final vaccine is 0.5705 with a virus model at a threshold of 0.4, suggesting a high antigenicity of our final vaccine. The AllergenFP 1.0 server and AllerTOP 2.0 server^56^ predict the final vaccine and its every subunit to be non-allergenic (see Table 9). The solubility of the final vaccine and its every subunit is evaluated by SolPro^57^ and Protein-sol server^58^. The predicted values suggest that our final vaccine and its every subunit have good solubility (see Table 9).

**Table 9:** Antigenicity, allergenicity and solubility Evaluation Results. NA: non-allergen. Higher Vaxijen and Antigen Pro scores suggest higher antigenicity. Higher SolPro and Protein-sol scores suggest higher solubility.

Vaccine subunits	Vaxijen score	Antigen pro score	AllerTOP result	Allergen FP result	Solubility by SolPro	Solubility by protein-sol
Adjuvant	0.7447	0.8205	NA	NA	0.7568	0.716
Subunit 1	0.2486	0.4137	NA	NA	0.5890	0.684
Subunit 3	0.4791	0.5923	NA	NA	0.8113	0.660
Subunit 4	0.3891	0.7364	NA	NA	0.6242	0.608
Subunit 5	0.4757	0.4768	NA	NA	0.7819	0.686
Subunit 10	0.3615	0.6256	NA	NA	0.6023	0.652
Subunit 13	0.8318	0.4032	NA	NA	0.9114	0.730
Subunit 18	0.2449	0.3076	NA	NA	0.9928	0.742
Subunit 19	0.3605	0.4991	NA	NA	0.7831	0.636
Subunit 23	0.6713	0.7355	NA	NA	0.6891	0.640
Subunit 24	0.4012	0.5211	NA	NA	0.9747	0.545
Subunit 25	0.6035	0.7433	NA	NA	0.6425	0.947
Final Vaccine	0.5705	0.8814	NA	NA	0.7555	0.723

### Toxicity and physicochemical properties analysis

The vaccine must not have toxicity potential and the physicochemical properties are also important to evaluate how the vaccine interacts with the environments^59^. We use the ToxinPred server^60^ to predict the toxicity. Other physicochemical properties, including hydropathicity, charge, half-life, instability index, pI (theoretical isoelectric point value) and molecule wheight, are predicted by ExPASy ProtParam Tool^61^. For the whole final vaccine sequence and the adjuvant sequence, we use the protein screening mode in the ToxinPred server to check all its overlapping peptides with length no more than 50 aa. The whole vaccine and the adjuvant do not contain any toxic part peptide. Other subunits and the 6xHis tag are checked by the SVM prediction mode in the ToxinPred server and all the subunits and the 6xHis tag are non-toxicity. The hydropathicity value of the final vaccine is predicted to be − 0.521. This negative value suggests that our final vaccine is hydrophilic in nature and can interact with water molecules easily^62^. The charge is 37.00; this value will decrease in alkaline environment so usually it is better if the charge values are positive. The half-life of the final vaccine is predicted to be 30 h in vitro and > 20 h in vivo. An Instability Index of 34.01 is predicted; this being less than 40 threshold value suggests that our final vaccine is stable. The pI of the final vaccine is calculated to be 9.75, which is an alkaline value, indicating its highly basic existence in nature. The molecular weight of the final vaccine is calculated to be 76 kDa. We also check the toxocity and physicochemical properties of every subunit and the results are shown in Table 10.

**Table 10:** Toxicity and physicochemical properties prediction results. NT: none-toxicity. We use the protein screening mode in the ToxinPred server to check the overlapping peptides in the final vaccine and adjuvant sequence and they do not contain any toxic peptide. For the rest subunits, we directly use the SVM based prediction to predict their toxicity.

	Toxicity	Hydropathicity	Charge	Half-life (in vitro)	Half-life (in vivo)	Instability index	Stability	pI	Mol. weight
Final vaccine	No toxic part	− 0.521	37.00	30 h	> 20 h	34.01	Yes	9.76	76,428.68
Adjuvant	No toxic part	− 0.679	28.00	30 h	> 20 h	38.94	Yes	10.30	30,396.93
Subunit 1	NT	− 0.510	3.00	7.2 h	> 20 h	34.35	Yes	9.99	3465.91
Subunit 3	NT	− 0.670	0.00	1.9 h	> 20 h	45.82	Yes	5.84	3277.00
Subunit 4	NT	− 0.880	0.50	5.5 h	3 min	69.83	No	6.75	3668.46
Subunit 5	NT	− 0.170	2.50	1.1 h	3 min	18.96	Yes	9.40	3545.56
Subunit 10	NT	− 1.053	3.00	1.4 h	3 min	7.15	Yes	9.71	3635.55
Subunit 13	NT	− 0.010	− 1.0	20 h	30 min	1.99	Yes	3.80	3095.51
Subunit 18	NT	− 0.897	0.00	1 h	30 min	25.35	Yes	6.31	3518.40
Subunit 19	NT	− 0.183	1.00	5.5 h	3 min	67.50	No	8.43	3348.34
Subunit 23	NT	− 0.050	3.00	5.5 h	3 min	38.38	Yes	9.20	3307.31
Subunit 24	NT	− 0.150	− 0.50	100 h	> 20 h	17.10	Yes	5.33	3548.92
Subunit 25	NT	− 0.450	− 1.50	1.1 h	3 min	24.99	Yes	7.75	3283.07
6xHis Tag	NT	− 3.20	0.00	3.5 h	10 min	8.33	Yes	7.21	840.86

### Secondary structure prediction

**Figure 5:**
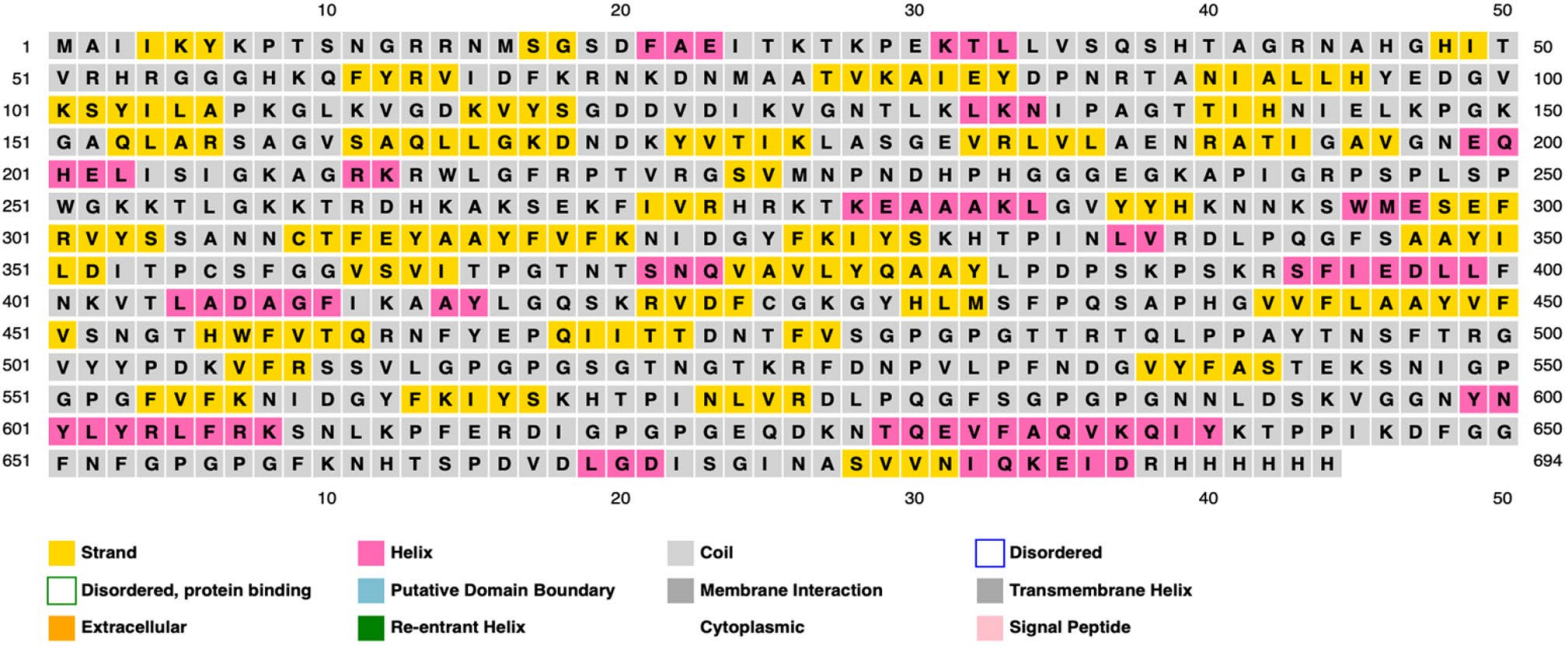
Graphical Representation of the Secondary Structure Features. The alpha helix residues are in pink, the beta strand residues are in yellow and the coil residues are in grey. The predicted secondary stucture indicates that the final vaccine constitutes 10.8% alpha helix, 24.6% beta strand, and 64.6% coil, respectively.

**Figure 6:**
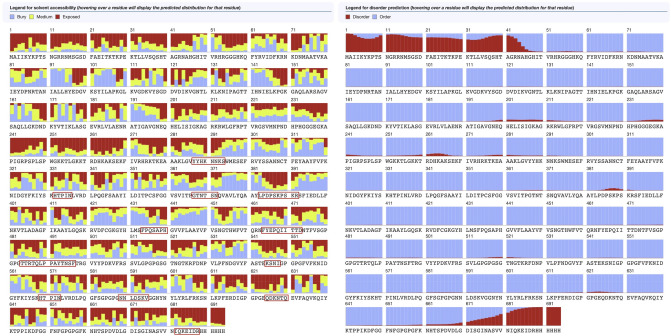
Solvent Accessibility and Disorder Regions Prediction Results. In the solvent accessibility prediction results, the red color represents the exposed residues, the yellow color represents the medium exposed residues and the blue color represents the buried residues. The peptides marked in red boxes are B-cell epitopes. The prediction results show that the B-cell epitopes in the final vaccine have good surface accessibility and also they are not close to each other. In the disorder regions prediction results, the ordered regions are in blue while the disordered regions are in red. A total of 60 residues (8%) are in disordered regions, showing good order in structure.

We use PSIPRED^63^ to generate the secondary structure of our final vaccine. Graphical representation of the secondary structure features are shown in Fig. 5. The predicted secondary stucture indicates that the final vaccine constitutes 10.8% alpha helix, 24.6% beta strand, and 64.6% coil. The solvent accessibility (ACC), and disorder regions (DISO) are predicted by RaptorX Property server^64,65^ (see Fig. 6). Among the 694 amino acid residues in our final vaccine, 44% are predicted to be exposed, 27% medium exposed, and 27% are predicted to be buried. The peptides marked in red boxes in Fig. 6 are the B-cell epitopes, showing good surface accessibility and they are not close to each other. A total of 60 residues (8%) are predicted to be located in disordered regions.

### Vaccine 3D structure modeling

**Figure 7:**
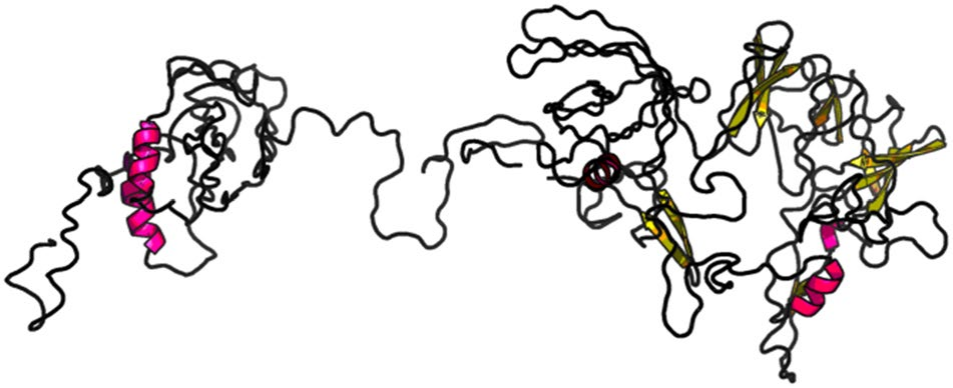
Vaccine 3D Structure Modeling by RaptorX based on the template with PDB ID 3j3vC. All the 694 amino acids in the final vaccine are modeled. The P-value of this model is 4.13 × 10 − 14 and this very low value indicates high quality of this 3D model. The unnormalized Global Distance Test (uGDT) score of this model is 506 (> 50), indicating good absolute model quality.

We use the RaptorX server^66^ to build the 3D structure models of our final vaccine. The protein structure with PDB ID 3j3vC is predicted by RaptorX to be the best template, based on which this server constructs the 3D structure model of our final vaccine (see Fig. 7). In this model, 100% (694) amino acids in the final vaccine are modeled in four domains. The P-value quantifies the likelihood of the predicted model being worse than other models generated randomly. The P-value for this model is calculated to be 4.13 × 10^−14^, which is a very low value, suggesting high quality of this 3D model. The unnormalized Global Distance Test (uGDT) score measures the absolute model quality. The overall uGDT score is predicted to be 506 and being greater than the 50 threshold value for a protein with more than 100 amino acid residues indicates that the 3D model of our final vaccine is good for further refinement.

### Vaccine 3D structure refinement

**Figure 8:**
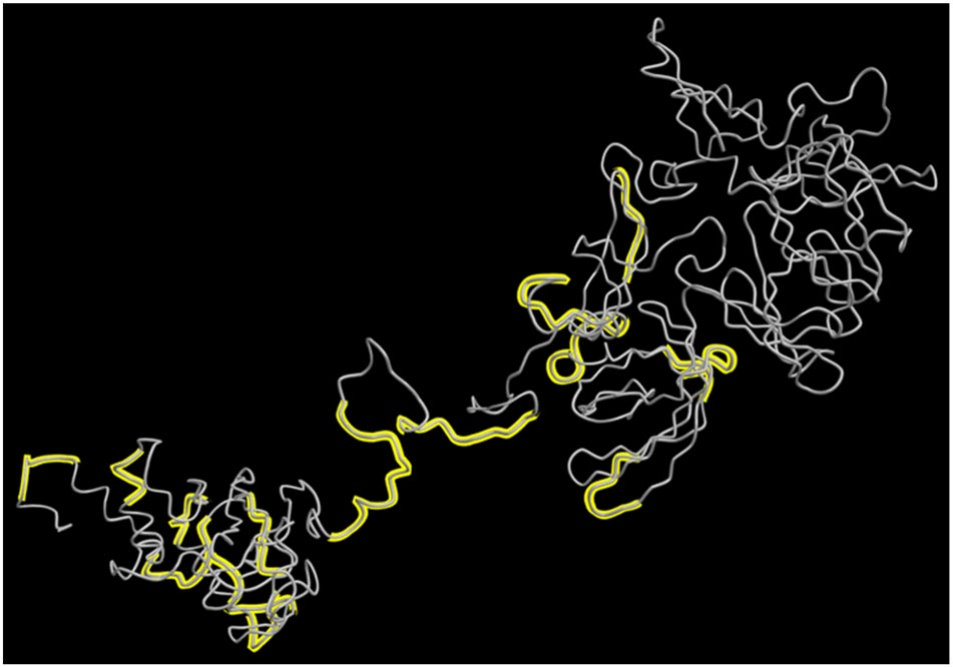
Refined Vaccine 3D Structure Model by GalaxyRefine. This model has a Global Distance Test—High Accuracy (GDT-HA) score of 0.900, a Root Mean Square Deviation (RMSD) score of 0.580, a MolProbity score of 2.618, a clash score of 33.5 and a Ramachandran plot score of 87.5%, showing great overall model quality. The B-cell epitopes in this final vaccine 3D model are highlighted in yellow.

We use GalaxyRefine server^67^ to refine the 3D structure model of our final vaccine. Among the 5 refined models predicted by GalaxyRefine, we choose the Model 2 shown in Fig. 8 as our final vaccine model based on its model quality scores (see Table 11). The predicted B-cell epitopes are highlighted in yellow, showing good surface accessibility. Global Distance Test—High Accuracy (GDT-HA) score measures the similarity between two protein structures. The GDT-HA score between this refined model and the initial model reaches a high value of 0.900, indicating that they have high similarity. The distance between atoms is measured by the Root Mean Square Deviation (RMSD) score. Lower RMSD value suggests better stability and usually an RMSD score ranges between 0 and 1.2 is acceptable. This model has an RMSD score of 0.580. Such RMSD score indicates stable protein structure. Molprobity score reflects the crystallographic resolution of the model. The MolProbity score of our identified vaccine model is 2.618, which is much lower than the initial model, showing that the refinement has lowered the critical errors of the 3D model. The Clash Score reflects the number of unfavorable all-atom steric overlaps and the refinement reduced the clash score of the model from 137.8 to 33.5, improving the model stability to a high level. The Ramachandran plot score represents the size of energetically favoured regions and usually a value greater than 85% is acceptable. The Ramachandran plot score has been improved from 78.3 to 87.5% by the refinement. The quality scores of the refined model shows good overall quality.

**Table 11:** Quality scores of the models predicted by GalaxyRefine.

Model	GDT-HA	RMSD	MolProbity	Clash score	Rama favored
Initial model	1.0000	0.000	4.229	137.8	78.3
Model 1	0.8941	0.588	2.703	33.4	87.5
Model 2	0.9000	0.580	2.618	33.5	87.5
Model 3	0.8922	0.590	2.657	33.9	87.2
Model 4	0.8966	0.583	2.698	33.7	87.3
Model 5	0.8977	0.582	2.632	34.0	87.5

### Vaccine 3D structure validation

**Figure 9:**
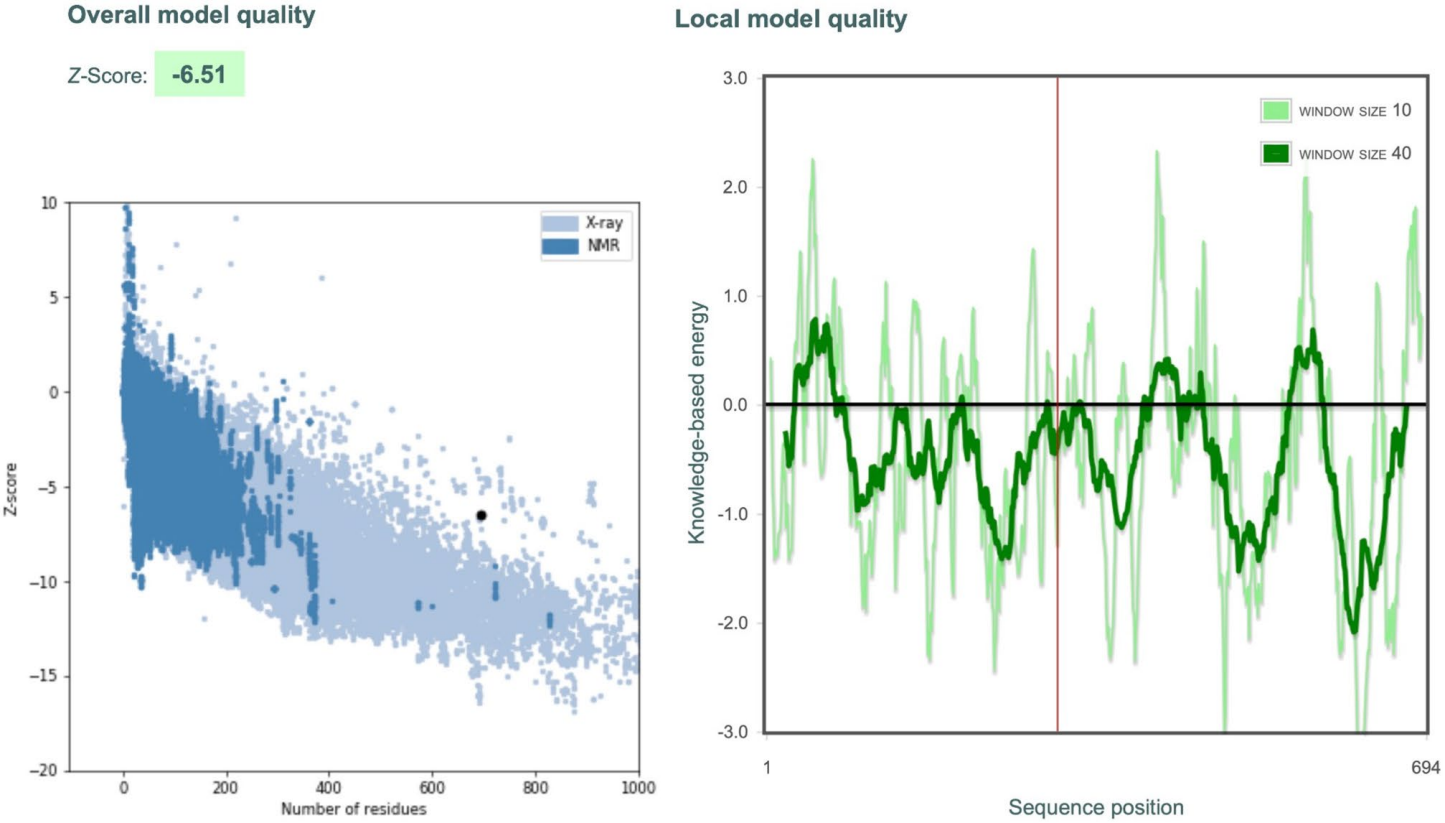
Vaccine 3D Structure Validation by ProSA-web. The Z-score of the refined model is -6.51 which is lying inside the score range. ProSA-web also plots the residues scores to check the local model quality and the negative values suggest no erroneous parts of the model structure.

We use ProSA-web^68^ to validate the overall model quality of the refined final vaccine model. ProSA predicts a Z-score of -6.51 (see Fig. 9) for the refined model, which is lying inside the score range of the comparable sized native proteins, indicating good overall model quality. ProSA also checks the local model quality and the residue scores are plotted in Fig. 9. Negative values suggest no erroneous parts of the model structure. We also use RAMPAGE server to do the Ramachandran plot analysis and it reveals a Ramachandran plot score of 87.5%, which is consistent with the results of GalaxyRefine.

### Conformational B-cell epitope prediction

**Figure 10:**
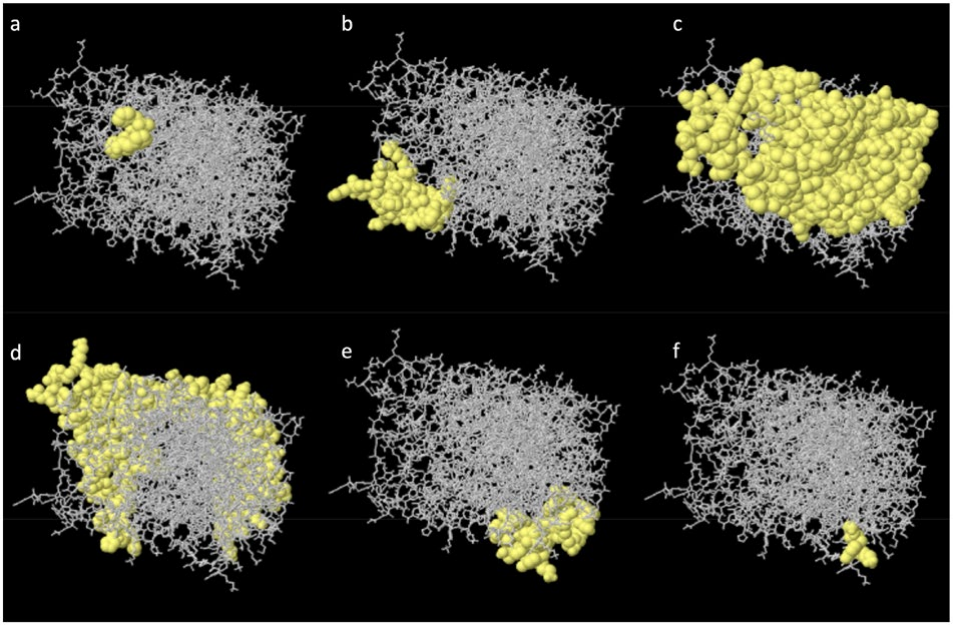
The 3D model of the 6 predicted conformational B-cell epitopes in the refined final vaccine structure. The yellow parts are the conformational B-cell epitopes and the grey parts are the rest of the residues. **(a)** 3 residues with a score of 0.963. **(b)** 30 residues with a score of 0.757. **(c)** 167 residues with a score of 0.711. **(d)** 161 residues with a score of 0.688. **(e)** 23 residues with a score of 0.59. **(f)** 3 residues with a score of 0.531.

The structure and folding of the new protein can result in new conformational B-cell epitopes which requires additional predictions. We use ElliPro server^69^ to predict the conformational B-cell epitopes in the refined 3D model. The ElliPro server predicts 6 new conformational B-cell epitopes which involved 387 residues with scores ranging from 0.531 to 0.963. The detailed 3D model and information of those 6 epitopes are shown in Fig. 10.

### Codon optimization and in silico cloning

**Figure 11:**
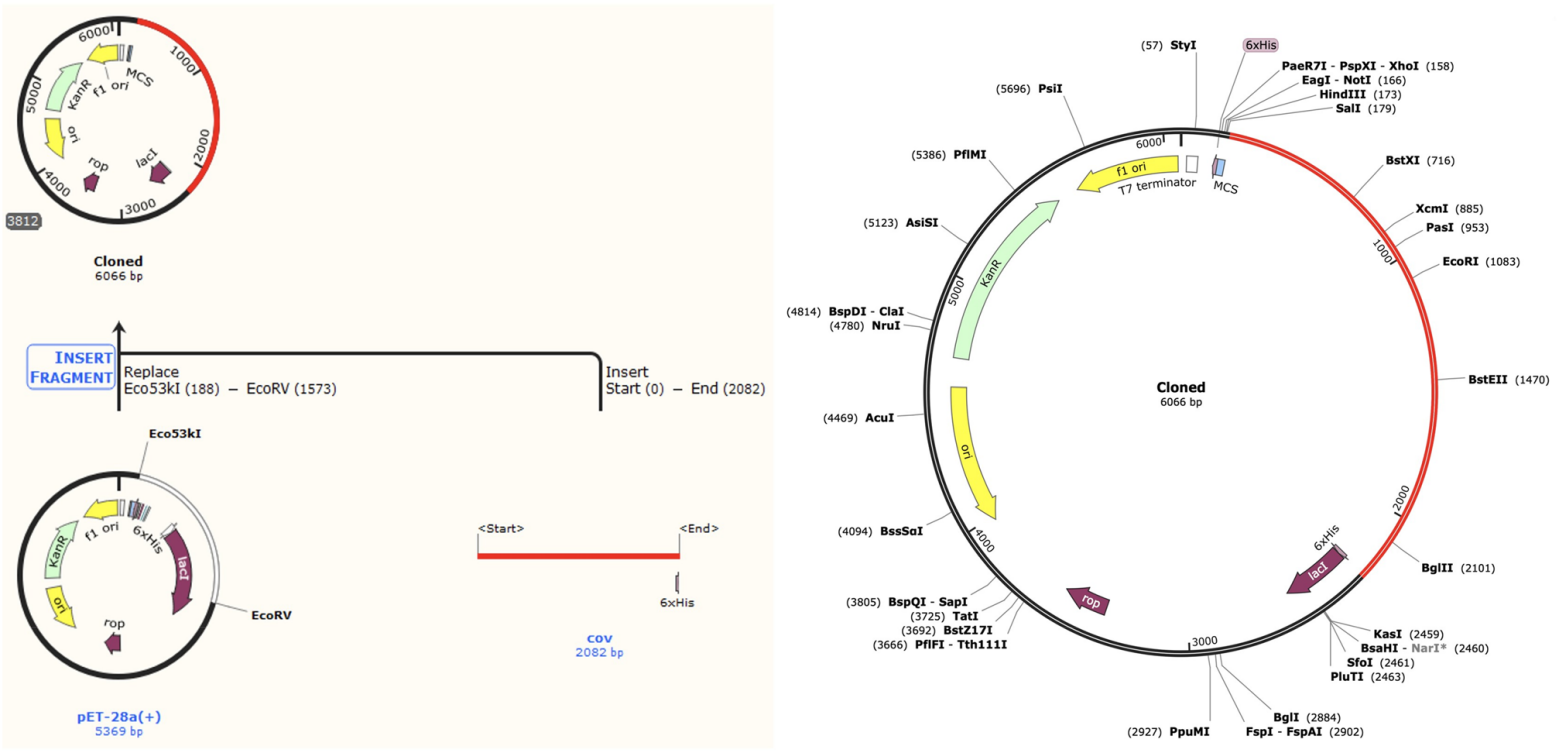
Final Vaccine in silico cloning into the pET28a( +) vector. The codon sequence of the final vaccine is in red, which is a 2082 bp gene sequence generated by the JCat server. The pET28a( +) expression vector is in black. The codon sequence is inserted between Eco53KI (188) and EcoRV (1573), forming a clone with a toal length of 6066 bp. This image was created by SnapGene 5.1.5 software (from Insightful Science; available at https://www.snapgene.com).

We analyze the cloning and expression efficiency and optimize the codon usage of vaccine construct in E. coli (Escherichia coli) strain K12) by Java Codon Adaptation Tool^70^. The length of the optimized codon sequence is 2082 nucleotides. Its Codon Adaptation Index (CAI) is 0.997, and the average GC content is 50.73%, indicating a great potential of good expression of the final vaccine in the E. coli host. After the optimization, we use the SnapGene tool to insert the codon sequences into pET28a( +) vector for cloning^71^ (see Fig. 11). The codon sequence of the final vaccine is presented in red, which is the 2082 bp gene sequence generated by the JCat server. The pET28a( +) expression vector is in black. The codon sequence is inserted between Eco53KI (188) and EcoRV (1573), forming a clone with a total length of 6066 bp.

### Molecular docking

**Figure 12:**
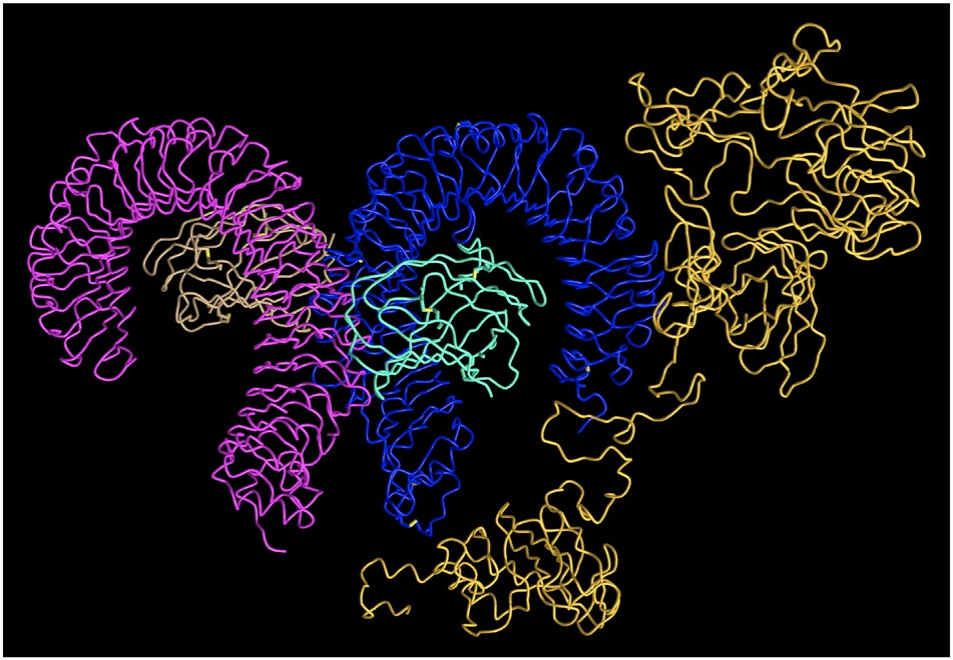
The docked complex of the vaccine model and the TLR4 immune receptor. The vaccine protein is in yellow and the rest of the residues is the TLR4 receptor. The lowest energy score of this complex model is -1311.5, indiating good binding affinity.

Molecular docking can evaluate the interactions between a ligand molecule and the receptor molecule to check the stability and binding affinity of their docked complex. Toll-like receptor 4 is an important human protein for pathogen recognition and immune response. Consequently, we choose TLR4 as the immune receptor to perform the molecular docking. We use the ClusPro 2.0 server^72^ to perform the molecular docking between the refined 3D model of our final vaccine and the TLR4 (PDB ID: 4G8A) immune receptor. Among all the generated docking model, we select the one with the lowest energy score of -1311.5 as the best docked complex, suggesting that the vaccine model occupies the receptor properly and indicating good binding affinity (see Fig. 12).

### Molecular dynamics simulation of the vaccine-receptor complex

**Figure 13:**
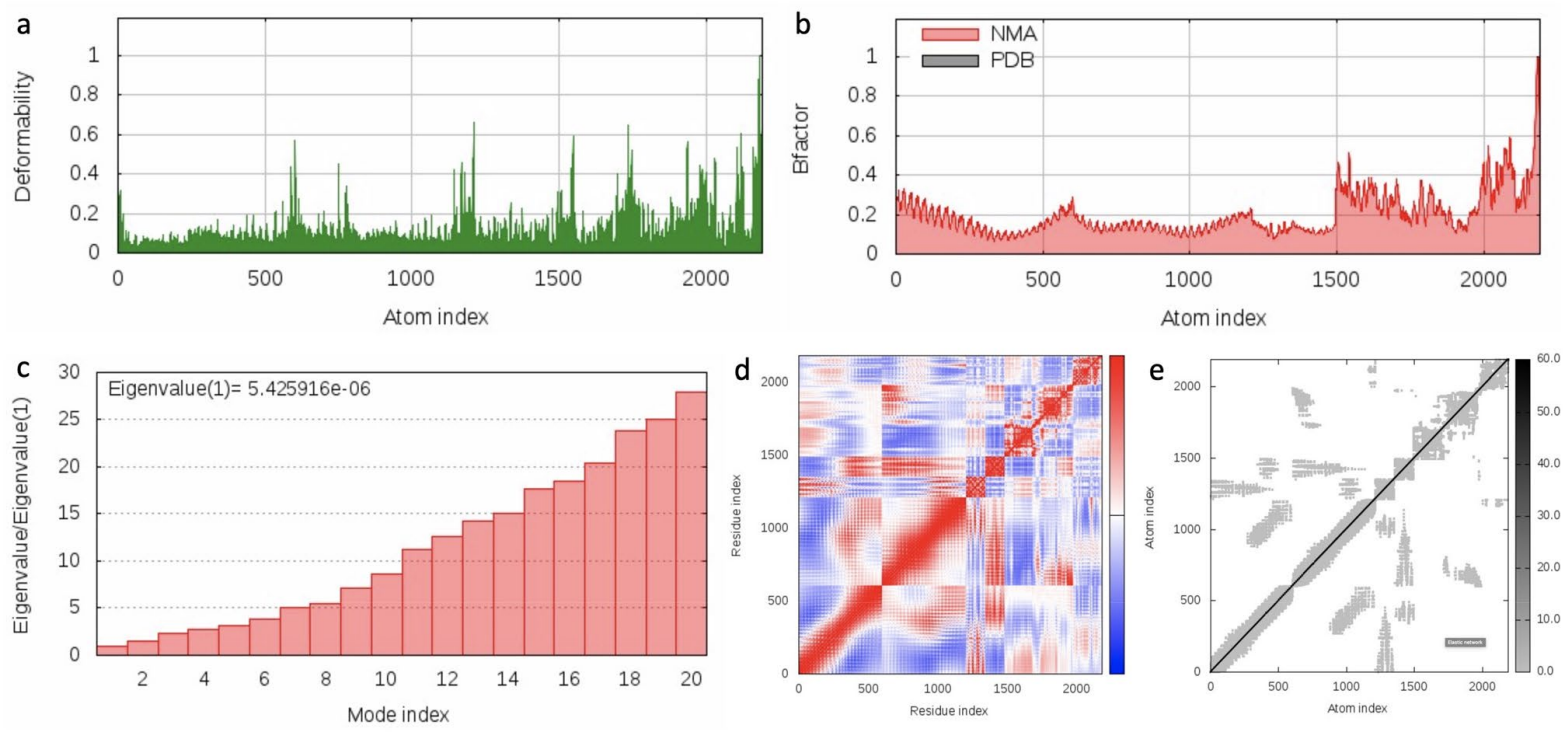
The molecular dynamics simulation of the vaccine-TLR4 docked complex. **(a)** Main-chain deformability simulation, the hinges are regions with high deformability. **(b)** B-factor values calculated by normal mode analysis, quantifying the uncertainty of each atom. **(c)** The eigenvalue of the docked complex, showing the energy required to deform the structure. **(d)** The covariance matrix between pairs of residues (red: correlated, white: uncorrelated, blue: anti-correlated). **(e)** The elastic network model, suggesting the connection between atoms and springs. The springs are more rigid if their greys are darker.

To evaluate the stability and physical movements of the vaccine-TLR4 docked complex^17,73^, we perform molecular dynamics simulation by the iMOD server^74^. The main-chain deformability is shown in Fig. 13a. The locations with hinges are regions with high deformability. The B-factor values calculated by normal mode analysis are proportional to root mean square (see Fig. 13b). B-factor values quantify the uncertainty of each atom. Figure 13c presents the eigenvalues which are closely related to the energy required to deform the structure and the eigenvalue of the complex is 5.426 × 10^−6^. The covariance matrix between the pairs of residues is shown in Fig. 13d, indicating their correlations (red: correlated, white: uncorrelated, blue: anti-correlated). The elastic network model is shown in Fig. 13e, suggesting the connection between atoms and springs. The molecular dynamic simulation results suggest that our vaccine model is stable.

### RNA mutations

As the SARS-CoV-2 spreads all over the world, its RNA sequence is going through mutations, translating out different virus proteins. Such mutations can have influences on the epitope based vaccines, since a single amino acid difference can change the epitope prediction results. Therefore it is important to prove that the proposed final multi-epitope vaccine can tackle the mutations. With our DeepVacPred, we are also able to quickly examine the mutated protein sequences to search for new potential vaccine subunits.

The RNA sequence we use to translate the spike protein and design the vaccines is from Wuhan, which is the place of the original virus^35^. The RNA mutations lead to three most frequent changes in the spike protein area of the SARS-CoV-2 and each of the changes contains one amino acid change^75^. Table 12 shows the mutation details.

**Table 12:** Spike protein mutations. Occurrence is the number of isolates that showed the mutation. Region is the origin of the isolates.

Mutations	Occurrence	Regions
G476S	3	Washington
V483A	6	Washington
D614G	116	Washington, Los Angeles, New York, South America, Europe

The mutation at the 614aa in spike protein from D to G is the most frequent mutation with 116 known isolates^75^. This mutation is very common in many cities in North America. In Europe and South America the D614G mutation occurs in less than 10 isolates. This change has no influence on the final multi-epitope vaccine since it does not contain the 614aa of the spike protein. With DeepVacPred, we are also able to quickly check and identify whether the mutation can create new potential vaccine subunits. We input the mutated protein sequence into DeepVacPred and the predicted subunits are the same as the original virus.

At 476aa in spike protein there is a frequent mutation from G to S, which occurs in 3 isolates from Washington DC^75^. This mutation has no influence on the final multi-epitope vaccine since it does not contain the 476aa of the spike protein. We input the mutated protein sequence into DeepVacPred and the predicted subunits are the same as the original virus.

At 483aa in spike protein there is a frequent mutation from V to A, which occurs in 6 isolates from Washington DC^75^. This mutation has no influence on the final multi-epitope vaccine since it does not contain the 483aa of the spike protein. We input the mutated protein sequence into DeepVacPred and the predicted subunits are the same as the original virus.

In conclusion, our designed multi-epitope vaccine can tackle the current RNA mutations of the coronavirus. The current RNA mutations of the coronavirus create no new potential vaccine subunits.

## Discussion

In silico vaccine design has high value of efficacy and it strongly emphasizes the multi-epitope in the vaccine peptides. In this study, we develop DeepVacPred, an efficient vaccine subunit sieving framework, that exploits an AI-based approach to rapidly select 26 potential vaccine subunit candidates, introducing a new way for achieving a much higher speed and efficiency in in silico vaccine design. The goal is to directly predict the potential vaccine subunit sequence without the need to do a large number of different predictions, as well as to evaluate and select the predicted results manually. With this AI-based framework, we are able to skip at least 95% of unnecessary predictions and let the computer analyze and select the best vaccine subunits for us. DeepVacPred predicts the 26 vaccine subunits within less than a second, which enables us to skip the most time consuming part of the in silico vaccine design. With DeepVacPred, a researcher can construct a multi-epitope vaccine for a new virus and validate its quality within an hour.

This approach can be further developed by enhancing the complexity and coverage of the dataset. In this study, we selected a part of known epitopes and protective antigens to form the dataset and use it for training the DNN architecture. We use the simple bridging of one B-cell epitopes and one T-cell epitopes. With a more comprehensive dataset and more possibilities of epitope combinations, we will be able to develop a better, more comprehensive and quicker vaccine design tool. In spite of limited available datasets, the current framework can still deal with most of the situations now and provide an efficacious vaccine design.

The application of AI, and DNN methodology in particular, to protein sequences classification shows great potential. Most of the online tools rely on the SVM learning approaches. In the highly popular protective antigens prediction tool Vaxijen^34^, the AUC of the ROC curve can only reach 0.743, which cannot perform very accurate predictions. The dataset to train Vaxijen only contains 200 proteins, so it becomes more time consuming and challenging to rely on the SVM model with increasing number of discovered protective antigens. Consequently, the proposed DeepVacPred proves that DNN can perform a very accurate prediction with over 700,000 different proteins in the dataset.

This study eventually results in a novel multi-epitope vaccine with a length of 649aa against the SARS-CoV-2. It contains an adjuvant, 11 subunits with 16 B-cell epitopes, 82 CTL epitopes and 89 HTL epitopes. It shows good antigenicity, population coverage and good physichochemical properties and structures, providing great potential for the next step COVID-19 vaccine design with actual experiments and clinical studies.

Furthermore, we trace the RNA mutations of the SARS-CoV-2 virus. Basically, the RNA mutations can result in one amino acid change in the spike protein or other related proteins. The proposed vaccine design framework can also tackle the three most frequently observed mutations as well as it can be extended to deal with other potentially unknown mutations. The investigation on the RNA mutations also proves the high efficiency of our DeepVacPred. As future work, we will investigate novel AI algorithms and architectures capable of constructing multi-epitope vaccine designs that can overcome the unknown unknowns of viruses evolution.

## Methods

### DNN design and training in DeepVacPred framework

Each data input to the DNN architecture is a sequence with a length of 45 vectors which is converted from its protein sequence by Z-descriptors^31^ and ACC transformation^32^. Convolutional Neural Network (CNN) exhibits good performance to identify and process such vectors while multi-layer linear neural network is broadly connected to the ouput layer of the CNN, forming a complex DNN to enhance the classification ability. Hence, our DNN is constructed by the following layers and the parameters of each layer is decided using a random search to obtain high accuracy while maintaining good computing speed:

i. CNN, in channels = 1, out channels = 16, kernel size = 3, stride = 2, padding = 1, Tanh function;

ii. CNN, in channels = 16, out channels = 16, kernel size = 3, stride = 2, padding = 1, Tanh function;

iii. CNN, in channels = 16, out channels = 1, kernel size = 3, stride = 2, padding = 1, Tanh function, average pooling;

iv. Linear, in features = 32, out features = 64 , Tanh function;

v. Linear, in features = 64, out features = 32, Tanh function;

vi. Linear, in features = 32, out features = 16, Tanh function;

vii. Linear, in features = 16, out features = 2, Sigmoid function.

The hyper-parameters of the DNN training are listed below. The selected hyper-parameter values are marked in bold. We choose the hyper-parameters with good accuracy while maintaining good computing speed by using a random search.

i. Learning rate: [0.0001, 0.0005, **0.001**, 0.0015, 0.002];

ii. Optimizer: [SGD, RMSProp, **Adam**];

iii. Epochs: [2000, 4000, **6000**, 8000, 10000];

iv. Batch size: [1024, 2048, **4096**, 8192].

### Linear B-cell epitopes prediction

We use four popular server to predict the linear B-cell epitopes on each vaccine subunit candidates. (1) BepiPred-2.0 web server (http://www.cbs.dtu.dk/services/BepiPred/). BepiPred is a reliable machine learning based tool trained by random forest algorithm and its training dataset covers a large number of known linear B-cell epitopes from the IEDB database^25^. (2) ABCpred (http://www.imtech.res.in/raghava/abcpred/). ABCPred applies recurrent neural network to the classification of epitopes and non-epitopes to improve the accuracy^39^. (3) SVMTrip (http://sysbio.unl.edu/SVMTriP/). SVMTrip uses support vector machine to predict antigenic epitopes and its AUC reaches a value of 0.702^38^. (4) BCPreds (http://ailab.ist.psu.edu/bcpred/). BCPreds is also based on SVM model with an AUC value of 0.758 and its prediction relies on kernel methods^40^. The B-cell surface accessibility is checked by IEDB Emini tool^42^.

### Cytotoxic T lymphocytes (CTL) epitopes prediction

We use NetMHCpan 4.1 server (http://www.cbs.dtu.dk/services/NetMHCpan/) to predict the CTL epitopes on each vaccine subunit candidates. We predict the CTL epitopes with a length of 9aa. All the parameters are set at default. NetMHCpan predicts peptide binding to any MHC Class I molecule of known sequence using artificial neural networks (ANNs) which is trained on a combination of more than 850,000 quantitative Binding Affinity (BA) and Mass-Spectrometry Eluted Ligands (EL) peptides, providing reliable prediction results^43^.

### Helper T lymphocytes (HTL) epitopes prediction

We use NetMHCIIpan 4.0 server (http://www.cbs.dtu.dk/services/NetMHCIIpan/) to predict the HTL epitopes on each vaccine subunit candidates. We predict the HTL epitopes with a length of 15aa. All the parameters are set at default. NetMHCIIpan predicts peptide binding to any MHC II molecule of known sequence using artificial neural networks (ANNs) which is trained on an extensive dataset of over 500,000 measurements of Binding Affinity (BA) and Eluted Ligand mass spectrometry (EL), covering the three human HLA-DR, HLA-DQ and HLA-DP alleles, providing reliable prediction results^46^.

### Multi-epitope vaccine construction

In this section, the BLASTp screening is done by the Uniprot server (https://www.uniprot.org/blast). BLASTp can identify similar regions between two sequences.

### Worldwide human population coverage analysis

The worldwide human population coverage of each subunit is evaluated by IEDB population coverage analysis tool (http://tools.iedb.org/population/). The evluation is done on the worldwide human population.

### Antigenicity, allergenicity and solubility evaluation

The antigenicity of the final vaccine and its every subunit is predicted by VaxiJen 2.0 server (http://www.ddg-pharmfac.net/ vaxijen/VaxiJen/VaxiJen.html) and AntigenPro server (http://scratch.proteomics.ics.uci.edu). Vaxijen is based on auto cross covariance (ACC) transformation of protein sequences into uniform vectors of principal amino acid properties^34^. Antigenpro is a sequence-based, alignment-free and pathogen-independant predictor of protein antigenicity^55^. The allergenicity of the final vaccine and its every subunit is checked by AllergenFP 1.0 server (http://ddg-pharmfac.net/AllergenFP/) and AllerTOP 2.0 server (https://www.ddg-pharmfac.net/AllerTOP/). AllergenFP and is a binary classfier between allergens and non-allergens. The dataset is described by five E-descriptors and the strings are transformed into uniform vectors by auto-cross covariance (ACC) transformation^76^. AllerTop is also based on ACC transformation and E-descriptors^56^. The solubility is evaluated by SolPro server (http://scratch.proteomics.ics.uci.edu) and Protein-sol server (https://protein-sol.manchester.ac.uk). SolPro is an SVM based tool to predict the solubility of a protein sequence with an overall accuracy of over 74% estimated by tenfold cross-validation^57^. Protein-sol is based on available data for Escherichia coli protein solubility in a cell-free expression system^58^.

### Toxicity and physicochemical properties analysis

The toxicity of the final vaccine and its every subunit is predicted by ToxinPred server (http://crdd.osdd.net/raghava/toxinpred/). TonxinPred is based on SVM model to classify toxicity and non-toxicity. The dataset used in its method consists of 1805 toxic peptides (≤ 35 residues)^60^. The physicochemical properties of the final vaccine and its every subunit is predicted by ExPASy ProtParam server (https://web.expasy.org/protparam/). The physicochemical properties include hydropathicity, charge, half-life, instability index, pI (Theoretical isoelectric point value) and molecule wheight^61^.

### Secondary structure prediction

PSIPRED is used for the secondary structure prediction of our final vaccine (http://bioinf.cs.ucl.ac.uk/psipred/). PSIPRED incorporates two feed-forward neural networks which perform an analysis on output obtained from PSI-BLAST (Position Specific Iterated—BLAST). It achieves an average Q3 score of 81.6%, which can achieve accurate secondary structure prediction^63^. We also use RaptorX Property web server (http://raptorx.uchicago.edu/StructurePropertyPred/predict/) to predict the solvent accessibility (ACC) and disorder regions (DISO). RaptorX employs an emerging machine learning model called DeepCNF (Deep Convolutional Neural Fields) to predict secondary structure (SS), solvent accessibility (ACC), and disorder regions (DISO) simultaneously^65^.

### Vaccine 3D structure modeling

The 3D model of the final vaccine is constructed by RaptorX server (http://raptorx.uchicago.edu/ContactMap). RaptorX provides distance-based protein folding powered by deep learning. This server was officially ranked 1st in contact prediction in both CASP12 and CASP13 and initiated the revolution of protein structure prediction by deep learning^66^.

### Vaccine 3D structure refinement

The 3D model built by RaptorX server is refined by GalaxyRefine (http://galaxy.seoklab.org/cgi-bin/submit.cgi?type=REFINE). GalaxyRefine first rebuilds side chains and performs side-chain repacking and subsequent overall structure relaxation by molecular dynamics simulation. According to the CASP10 assessment, the GalaxyRefine server method performed the best in improving local structure quality^67^ The quality of the refined model is evaluated in terms of its GDT-HA socre, RMSD score, Molprobity score, clash score and Ramachandran plot score.

### Vaccine 3D structure validation

The final refined 3D model of our final vaccine is validated by ProSA-web server(https://prosa.services.came.sbg.ac.at/prosa.php). ProSA calculates an overall quality score for a specific input structure. If this score is outside a range characteristic for native proteins the structure probably contains errors. A plot of local quality scores points to problematic parts of the model which are also highlighted in a 3D molecule viewer to facilitate their detection^68^.

### Conformational B-cell epitope prediction

The conformational B-cell epitopes in the refined final vaccine 3D structure model are predicted by the ElliPro Server (http: //tools.iedb.org/ellipro). ElliPro is based on the geometrical properties of protein structure. Among the current conformational B-cell epitope prediction tools, ElliPro has the best AUC score of 0.732, which is a very reliable tool for identifying antibody epitopes in protein antigens^69^.

### Codon optimization and in silico cloning

Java Codon Adaptation Tool (JCat) server is used for codon optimization (https://urldefense.com/v3/__http://www.jcat.de/LIr3w8kk_Xxm!7wRJ08pRiYapODc_l0a3Lu91JwL-k63K5zWwthwiCfq_ctg6SmoWSkB2JxUzyRA). JCat adapts the codon usage to most sequenced prokaryotic organisms and selected eukaryotic organisms^70^. The optimized codon sequence is insert into pET28a( +) vector with SnapGene 5.1.5 software (from Insightful Science; available at https://www.snapgene.com).

### Molecular docking

The molecular docking is done by ClusPro 2.0 server (https://cluspro.bu.edu). ClusPro is a widely used tool for protein–protein docking. Docking with each energy parameter set results in ten models defined by centers of highly populated clusters of low-energy docked structures^72^. We choose TLR4 (PDB ID: 4G8A) as the immune receptor. We select the docked complex with the lowest energy score.

### Molecular dynamics simulation of the vaccine-receptor complex

The molecular dynamics simulation is done by iMOD server (iMODS) (http://imods.chaconlab.org). iMODS facilitates the exploration of such modes and generates feasible transition pathways between two homologous structures^74^. The iMOD server evaluates the protein stability by computing its internal coordinates through normal mode analysis (NMA). The stability of the protein is represented in terms of its main-chain deformability plot, B-factor values, eigenvalue, covariance matrix and elastic network model.

## Data Availability

We obtained the genome sequence and the spike protein sequence of SARS-CoV-2 from NCBI database (https://www.ncbi.nlm. nih.gov) with accession number MN908947 and protein ID QHD43416.1. The protein data we collected and processed to train the DeepVacPred is available on github.com (https://github.com/zikunyang/DCVST).

## Code availability

The code used for data generation and/or analysis in the study are available on github.com (https://github.com/zikunyang/DCVST).
